# High-throughput yeast engineering in biofoundries: towards autonomous and scalable synthetic biology

**DOI:** 10.1093/femsyr/foag003

**Published:** 2026-01-27

**Authors:** Juan P O Martinez, Robert E Speight

**Affiliations:** Advanced Engineering Biology Future Science Platform, Commonwealth Scientific and Industrial Research Organisation (CSIRO), Dutton Park, Brisbane, QLD 4102, Australia; School of Biology and Environmental Science, Queensland University of Technology, Brisbane, QLD 4000, Australia; Advanced Engineering Biology Future Science Platform, Commonwealth Scientific and Industrial Research Organisation (CSIRO), Dutton Park, Brisbane, QLD 4102, Australia; School of Biology and Environmental Science, Queensland University of Technology, Brisbane, QLD 4000, Australia

**Keywords:** biofoundry, synthetic biology, engineering biology, machine learning

## Abstract

High-throughput yeast engineering is being transformed by biofoundries that integrate automation, artificial intelligence (AI), and standardized workflows. This review examines how these facilities accelerate strain development through the Design-Build-Test-Learn (DBTL) cycle, with advances in genome editing, phenotypic screening, and predictive modelling. It highlights Australia’s involvement through the Australian Genome Foundry, Idea-BIO, and the CSIRO Biofoundiry and explores global efforts to overcome reproducibility and standardization challenges. Despite progress, key barriers remain, including protocol variability and integration of AI tools. We also highlight the opportunity for a shift toward autonomous, self-optimizing ‘self-driving labs’ that transition from DBTL to Design-Build-Deploy cycles. The future of yeast engineering depends not only on technological innovation, but also on the harmonization of international standards, data governance, and ethical safeguards. If fully realized, the convergence of robotics, AI, and synthetic biology will redefine yeast engineering, leading to step changes in strain performance for a variety of important products, thus enabling economic and sustainable biomanufacturing at scale.

## Teaser

This minireview provides a contemporary overview of the latest developments in biofoundry level yeast strain engineering in Australia and in general.

## Introduction

*Saccharomyces cerevisiae* has long been central to biotechnology due to its genetic tractability, robust industrial performance, and generally regarded as safe (GRAS) status (Wang et al. 2024). Recent advances in genome editing, systems biology, and high-throughput screening have expanded its role as a chassis to produce a wide range of valuable chemicals. A major focus now lies in optimizing strain performance by improving titre, rate, and yield (TRY) key parameters that determine industrial viability (Shi et al. 2025). The field is also rapidly broadening to include non-conventional yeasts, each offering specialized biosynthetic capabilities. Examples include *Komagataella phaffii* (*Pichia pastoris*) for heterologous protein production, *Yarrowia lipolytica* for lipid biosynthesis, and *Kluyveromyces marxianus* and *Scheffersomyces stipitis* for efficient bioethanol generation from diverse carbon sources (Borodina and Nielsen 2014, Kuivanen et al. 2018, Cai et al. 2019, J. Zhang et al. 2021, Wives et al. 2024).

To enable high-throughput (HTP) engineering of these diverse hosts, standardized genetic toolkits, transformation protocols, and high-throughput robotics enabled workflows are essential (Holowko et al. 2021, Koray et al. 2022). Technologies like CRISPR, modular cloning, *in vivo* recombination, and site-specific integrases support this expansion (Shen et al. 2024).

HTP yeast engineering, powered by biofoundries, accelerates the Design-Build-Test-Learn (DBTL) cycle through automation, AI, and advanced analytics (Nielsen and Keasling 2016, Chao et al. 2017, Dixon et al. 2023). These platforms enable rapid, reproducible strain optimization for applications in biomanufacturing, pharmaceuticals, and sustainability (Hillson et al. 2019). Robotics increases the number of strains that can be built and tested and ensure consistency across design, assembly, and cultivation, reducing the time from concept to strain (Holub and Agena 2023, Goold et al. 2025).

Despite these advances, challenges remain in standardization and reproducibility across labs (Lux et al. 2023). Integrating AI for strain selection and predictive modelling presents both opportunities and implementation hurdles (Pretorius 2017, Jervis et al. 2018). Recent advances emphasize that overcoming these hurdles will require the deep integration of AI with mechanistic metabolic models to enable accurate, data-driven strain design (Lu et al. 2024). Still, the convergence of multiomics, single-cell tools, and AI is transforming yeast engineering into a data-rich, precision field, as demonstrated by (Gao et al. 2022) and a recent preprint (Lyu et al. 2024).

The Global Biofoundry Alliance (GBA), established in 2019, fosters open standards and shared infrastructure among public foundries worldwide (Hillson et al. 2019). Australia plays a key role, with facilities like the Australian Genome Foundry, Idea-BIO, and the CSIRO Biofoundry contributing to global efforts in HTP engineering, data interoperability, and protocol harmonization (Dixon et al. 2023, Holub and Agena 2023, Goold et al. 2025, IDEA–Bio 2025). Regional collaborations include an Asia–Pacific Workshop, held in August 2023, which brought together 40 participants from 13 countries across industry, academia, and government to advance the harmonization of metrics and standards (Freemont 2024).

As biofoundries evolve, DBTL is increasingly shifting to Design–Build–Deploy (DBD), emphasizing rapid, AI–guided strain delivery (Dixon et al. 2022). Improvements in knowledge, modelling, and AI, fuelled by high–volume, high–quality omics, and high–throughput datasets, are markedly enhancing design predictability and accuracy, so that fewer iterative wet–lab tests are needed (e.g. ML models trained on multi–omics data now forecast pathway performance with greatly reduced experimental burden (Patra et al. 2023, Kugler and Stensjö 2024); deep integration of AI with genome–scale and enzyme–constrained models delivers more reliable TRY predictions (Kundu et al. 2024, Lu et al. 2024) retrieval–augmented LLM platforms automate knowledge extraction and design suggestions, speeding the transition from design to experiment (Li et al. 2025, Mao et al. 2025); and hybrid AI–automation systems cross–validate *in silico* predictions with in–line screening, ensuring robust strain delivery (Boob et al. 2024). Globally coordinated, AI–powered biofoundries represent the most scalable and strategic path forward for yeast strain engineering (Castaño-Cerezo et al. 2024). Establishing the infrastructure needed to support this next generation of biofoundries is critical. Australia offers several emerging models that demonstrate how investment in automation, standardization, and data interoperability can enable globally competitive synthetic biology platforms.

## Advances in yeast genome synthesis and synthetic biology toolkits

Australia is an important participant in the international synthetic biology community, with major advancements in yeast engineering and investment in automated research platforms (Pretorius 2017, Dixon et al. 2023). Integration of diverse scientific disciplines, coupled with progress in DNA sequencing and synthesis, underpins this growth. *Saccharomyces cerevisiae* has long served as a model for metabolic engineering, recently demonstrated by Australian-led innovations in isoprenoid biosynthesis. For example, an auxin-inducible protein degradation was implemented to conditionally redirect carbon flux toward mono- and sesquiterpene production, achieving up to 3.5 g l⁻¹ nerolidol by selectively depleting key enzymes (Lu et al. 2021). Similarly, combinatorial terpene pathway engineering and biosensor-guided strain optimization for high-value food and fragrance compounds were also explored (Bongers et al. 2020, Peng and Wei 2025). CRISPR has also been applied by Australian researchers to enhance yeast traits relevant to the wine and fermentation industries (Jagtap et al. 2017).

Beyond traditional engineering, yeast lipid droplets are being used for biocatalysis and biosensing (Suri et al. 2024). Synthetic genome minimization and biosafety strategies, like auxotrophy and toxin-antitoxin systems, are being explored to ensure environmental containment (Pretorius 2017).

Australian-led efforts have spearheaded two major chromosome-scale constructions within the global Yeast 2.0 (Sc2.0) project. Sc2.0 incorporated SCRaMbLE (Synthetic Chromosome Rearrangement and Modification by LoxP-mediated Evolution), an inducible Cre-loxPsym system that generates combinatorial genomic diversity through stochastic deletions, inversions and translocations. By placing loxPsym sites downstream of nonessential *loci*, SCRaMbLE enabled rapid exploration of genotype–phenotype landscapes, facilitating adaptive gains in pathway performance, stress tolerance or novel trait emergence (Shen et al. 2016). This capability has been applied not only to native chromosomes but also to synthetic neochromosomes (Erpf et al. 2025).

In 2022, Kutyna and colleagues at Macquarie designed and built a 211 kb pan-genome neochromosome by concatenating diverse sequence elements from eight industrial and environmental yeast strains, embedding 63 loxPsym sites to enable SCRaMbLE-mediated variability and providing new metabolic capabilities (Kutyna et al. 2022). More recently, in late 2024 the same team released the fully synthetic 903 kb synXVI, complete with optimized chunk termini, rebalanced tRNA content and corrected loxPsym insertions, demonstrating both high-fidelity assembly and iterative CRISPR-D-BUGS debugging to restore wild-type fitness (Goold et al. 2025).

Since its inception in 2006, the Sc2.0 consortium has pursued a bottom-up DBTL strategy to reengineer all 16 native chromosomes of *S. cerevisiae*, plus a synthetic tRNA neochromosome (Annaluru et al. 2014). Core design principles include replacement of TAG stop codons with TAA, removal of repetitive and nonessential elements, and integration of standardized PCRTags for assembly tracking. This ‘connecting-the-dots’ effort has unified computational design (BioStudio), hierarchical SwAP-In assembly, and centralized debugging workflows under the Yeast 2.0 banner (Pretorius and Boeke 2018).

High-throughput screening (HTS) systems are advancing recombinant protein production in *S. cerevisiae* and *K. phaffii* with work on promoter variants, terminators, and co-expression of folding chaperones like HAC1 and PDI improving yields (Peng et al. 2015, 2018, Navone et al. 2021). The advances for HTS also include yeast-based protein secretion biosensors for such organisms (Peng et al. 2021, Navone et al. 2023, Cleaver et al. 2024), and synthetic circuits for environmental and functional monitoring (Scott et al. 2018, 2020). Biosensor applications go beyond detecting proteins, as exemplified by the G-protein–coupled receptor biosensor detecting serotonin in *S. cerevisiae* (Saleski et al. 2024).

In sum, Australia’s strategic investments in synthetic genomics, metabolic engineering, automation, and AI have enabled important contributions to global synthetic biology innovation (Pretorius 2019, Dixon et al. 2023, Freemont 2024, Goold et al. 2025). However, to accelerate progress in synthetic biology, it will be essential to scale up the deployment of HTP platforms. When combined with robust automation, standardized workflows, and international collaboration, these systems can unlock faster DBTL cycles, enhance reproducibility, and support the translation of innovations into a design-built-deploy model and real-world applications.

## High-throughput phenotypic screening for complex traits

Synthetic biology’s shift to HTS and automation is driven by the DBTL cycle, which applies engineering principles to biological design (Kittleson et al. 2012, Carbonell et al. 2018, Pleiss 2024). The design phase uses computational tools like Benchling and Teselagento streamline construct planning, codon optimization, and constraint-based modelling (Nathan et al. 2012, Linshiz et al. 2014, Davies 2020, Fero et al. 2020).

The build phase leverages synthetic DNA, genome editing, and automation platforms such as AssemblyTron and AutoBiotech. Liquid-handling robots and acoustic droplet ejection systems reduce human error and reagent use whilst increasing throughput (Hadimioglu et al. 2016, Bryant et al. 2023).

In the test phase, yeast strains are evaluated using HTS platforms including microplate assays, FACS, multi-omics, and droplet microfluidics (Zhang et al. 2023, Lopez-Barbera et al. 2024, Shiraishi et al. 2024). Microfluidic systems encapsulate single cells in nanolitre droplets that behave as microbioreactors, enabling millions of miniaturized, parallel assays (Panwar et al. 2023) and link genotype to real-time reporter signals, enriching top variants via FACS (Zhang et al. 2023, Saleski et al. 2024).

The learn phase range from the use of simple tools, such as Excel and R, to advanced AI-driven models that predict genotype–phenotype relationships and refine experimental designs (Zhang et al. 2020). Robust laboratory information management systems (LIMS) and ML pipelines are increasingly critical for managing large datasets (Berezin et al. 2023, Zhang et al. 2025).

Modern biofoundries integrate these technologiesthrough automation, HTS, and data analysis evolving toward a DBD model focused on rapid translation to real-world applications (Dixon et al. 2022, Tu et al. 2022, Bryant et al. 2023). Table 1 provides an overview of the core hardware and software technologies enabling scalable HTP DBTL workflows in modern biofoundries.

**Table 1:** Key hardware and software technologies accelerating HTP DBTL workflows in synthetic biology. This table highlights representative tools used across the DBTL cycle in yeast synthetic biology. It includes automation platforms, microfluidic systems, liquid handling hardware, and computational tools that enable rapid strain prototyping, screening, and data-driven optimization. These technologies are central to modern biofoundries and the transition toward digitally integrated, deployable microbial solutions.

Workflow stage	Traditional approach	Automated biofoundry approach	Performance gain	References
**Design**	Manual CAD tools, limited rational design	AI-aided design using LLMs, PLMs, genome-scale metabolic models, BioCAD, SBOL, design Space exploration	+speed, +scope, +predictive power, +design diversity	(Gurdo et al. 2023, Ding et al. 2024, Ruan et al. 2024)
**Build**	Manual cloning, transformation	Automated modular DNA assembly (Golden Gate, MoClo, YTK) and genome editing (CRISPR/Cas, base editors) with robotics	+throughput, +modularity, +standardization, +multiplexing	(Chao et al. 2017, Koray et al. 2022, Gurdo et al. 2023)
**Test**	Plate-based assays, colony picking	HTS, microfluidics, FACS, omics, biosensors, single-cell analytics, automated data acquisition	+resolution, +data volume, +cell-level granularity, +speed	(Holland and Blazeck 2022, Martin et al. 2023)
**Learn**	Manual analysis, expert intuition	ML/AI integration (Bayesian optimization, deep learning, active learning), LIMS, cloud-based feedback loops	+predictivity, +cycle speed, +hypothesis generation	(Samek 2017, Jervis et al. 2018, Ding et al. 2024)
**Iteration time**	Weeks/months	Hours/days via closed-loop AI–robotics integration	×10–100 faster	(Gurdo et al. 2023, Martin et al. 2023)
**Standardization**	Informal protocols, lab-dependent/specific	Use of SBOL, SEVA, BioBricks, MIQE, modular toolkits, harmonized protocols across foundries	+reproducibility, +collaboration, +interoperability	(Bustin et al. 2009, Smolke 2009, Quinn et al. 2015, Chao et al. 2017, Freemont 2024)
**Automation level**	Manual, stepwise, pipetting robots	Fully autonomous self-driving labs (SDLs), digital twins, automated liquid handling, robotics integrated with AI agents	+consistency, +scalability, +parallelism, +24/7 uptime	(Gurdo et al. 2023, Martin et al. 2023, Kim 2025)
**Interoperability**	Siloed tools, format mismatches	Plug-and-play instrument modules via OPC UA, standard APIs, cloud-based coordination platforms	+flexibility, +device compatibility, +scale-out capability	(Freemont 2024, Li et al. 2024)
**Data infrastructure**	Spreadsheets, local servers	Integrated LIMS + data lakes, metadata tracking, real-time ML pipelines, FAIR-compliant architecture	+traceability, +ML-readiness, +multi-foundry learning	(Gurdo et al. 2023, Martin et al. 2023, Freemont 2024)
**Feedback mechanism**	Manual literature review, empirical design	Active learning loops, ML-guided optimization, LLM-based experiment planning, real-time sensor feedback	+exploration efficiency, +adaptability, +autonomy	(H. Yang et al. 2022, Ruan et al. 2024, Zhang et al. 2025)
**Sustainability**	Not evaluated routinely	Life cycle assessment (LCA), green chemistry metrics, low-waste microfluidics	+environmental compliance, +process efficiency, +public trust	(Asin-Garcia et al. 2024, Freemont 2024)
**Regulatory readiness**	Case-by-case, post hoc	Data-rich process logs, standardization for safety assessment, traceable workflows, reproducible synthetic routes	+approval speed, +regulatory transparency	(Freemont 2024)
**Biosafety & biosecurity**	Manual containment, SOPs	Automated risk classification, digital containment protocols, controlled access to genetic elements	+risk mitigation, +auditability, +compliance	(Hoffmann et al. 2023, Freemont 2024)
**Knowledge sharing**	Manual methods, internal notebooks	Open-source hardware/software, multi-foundry standard exchange formats	+community acceleration, +comparability	(Hillson et al. 2019, Gupta et al. 2024)

While HTP testing offers unparalleled data generation, its full potential is unlocked only when paired with computational tools capable of interpreting and using those data.

## AI, machine learning, and generative models in the DBTL cycle

Artificial intelligence (AI) and machine learning (ML) have reshaped synthetic biology by providing predictive modelling, accelerating experimental workflows, and integrating complex datasets. In yeast engineering, AI-driven pipelines can refine the Design–Build–Test–Learn (DBTL) cycle by guiding construct design, predicting performance, and optimizing metabolic pathways (Lee et al. 2023, Ramos et al. 2024). This section outlines how AI and generative models, combined with specialized software and high-throughput platforms, enhance DBTL accuracy, scope, and efficiency.

### Enhanced speed and predictive accuracy

Biofoundries increasingly incorporate AI solutions throughout DBTL to reduce trial-and-error, enable real-time feedback, and automate large-scale experimentation (Goshisht 2024, Zhang et al. 2025). Since every wet-lab assay carries significant cost and only a finite number of designs can be empirically tested, AI-driven in silico exploration of thousands of genetic configurations allows prioritization of high-confidence variants for validation, preserving scarce financial, temporal and material resources (Ramos et al. 2024). AI also assists in evaluating HTS data fidelity by flagging anomalies from robotic errors or instrument drift(Camacho et al. 2018), and supports standardization through domain-specific ‘Construction File’ (CF) formats that encode molecular biology operations and enable automated detection of design flaws and validation of workflows across labs (Ataii et al. 2023).

### Generative models in yeast engineering: PLMs and LLMs

Generative AI frameworks, including large language models (LLMs) and protein language models (PLMs), accelerate yeast engineering by automating pathway discovery and *in silico* design of genetic constructs (Radivojević et al. 2020, Cheng et al. 2023). ESM–2 predicts enzyme fitness metrics from sequence data and suggests mutations for downstream experimental validation in *S. cerevisiae* (Zhang et al. 2025), whilst LLM–driven pipelines could be adapted to draft yeast strain construction protocols and interface with liquid–handling robots or cell–free protein synthesis workflows (Herisson et al. 2024). Unsupervised generative models such as Generative Adversarial Networks or Variational Autoencoders could in principle explore combinatorial libraries of promoters, terminators and coding sequences to uncover novel regulatory elements that enhance metabolite production; an application that still awaits *in vivo* validation in yeast (Radivojević et al. 2020). As these tools mature, they could guide the design of bespoke biosynthetic pathways and tailor regulatory networks to the unique physiology of yeast hosts.

### AI-Driven data analysis and predictive modelling

AI–driven analytics underpin yeast metabolic engineering by transforming high–volume screening and multi–omics datasets into actionable design insights. Supervised methods such as support vector machines, random forests and deep neural networks, trained on integrated transcriptomic, metabolomic, and phenotypic profiles, enable accurate genotype–phenotype predictions that outperform linear models for complex traits (Goshisht 2024, Herisson et al. 2024). For example, deep learning frameworks have been used to predict metabolic flux distributions in engineered *S. cerevisiae*, closing the feedback loop in the ‘learn’ phase of DBTL and guiding subsequent strain optimization (Kim et al. 2020).

Hybrid mechanistic–ML pipelines embed genome–scale metabolic or kinetic models within transformer architectures to pinpoint pathway bottlenecks and recommend targeted gene interventions (Song et al. 2024). Retrosynthetic tools, RetroPath 2.0 and RetroBioCat, leverage curated enzyme databases alongside ML–guided route planning to propose multi–step biosynthetic pathways specifically optimized for yeast hosts (Finnigan et al. 2021, Yonet et al. 2024). Automated guide–RNA design with CRISPR–GPT has streamlined multiplexed genome by generating high–confidence editing libraries (Qu et al. 2024).

Underpinning these capabilities are robust data standards and tight integration with laboratory information management systems. Structured formats (e.g. SBOL) and ‘Construction File’ representations ensures seamless data exchange, automated error checking, and full provenance tracking across biofoundry workflows (Ataii et al. 2023, Cheng et al. 2023, Song et al. 2024, Vidal et al. 2025). A recent preprint underscores the importance of interpretability, traceability, and auditability in AI–driven experimentation, makes compliance an essential design consideration for any large–scale yeast engineering project (Boiko et al. 2023).

### Future prospects for ‘speed-to-strain’ engineering

As LLMs and generative models converge with robotic platforms, fully autonomous DBTL pipelines could drastically shorten design cycles (Ramos et al. 2024). Reduced trial-and-error and targeted combinatorial searches enable ‘speed-to-strain’ approaches, where AI prunes infeasible designs and proposes high-yield constructs, while improving scale-up decisions by factoring in real-time quality data (Yook and Alper 2025).

These integrated AI frameworks promise yeast engineering guided by flexible, multi-modal models that unify text, structure, and experimental data, shifting synthetic biology toward large-scale, autonomous engineering practices (Boiko et al. 2023, Cheng et al. 2023). Table 2 summarizes the main AI/ML tools used in yeast engineering with other relevant tools for automation that have not been validated in yeast systems are in Table S1.

**Table 2:** AI/ML tools used in yeast synthetic biology and their functions.

Tool/model	Type	DBTL role	Function/ application	Input/output	Yeast eng. application	Reference
ESM-2	Protein language model (LLM)	DL	Zero-shot fitness prediction of protein variants, design of enzyme libraries	Protein sequences Fitness likelihood scores	Predicts the impact of coding mutations on yeast‐expressed enzymes.	(Zhang et al. 2025)
Digital twins	Simulation/hybrid ML model	D	Predict pathway flux, simulate strain behaviour, virtual strain construction	Design specs → phenotype simulations	Simulates strain performance to rank designs before experiments.	(Gurdo et al. 2023)
Alphafold all atom/multimer	Structure prediction + ML	D	Predicts 3D structure of designed enzymes in new pathways, even conformed as multimers	Sequence → structure (PDB)	Predicts structures of engineered proteins before expression.	(Evans et al. 2021, Hou et al. 2025)
AUTOGPT + LIMS	Agent framework + LLM	BL	Self-guided agents for planning, executing, and refining biofoundry tasks, including multigene construct synthesis	Text prompt + context → experimental plan	Orchestrates end–to–end strain construction workflows.	(Zhang et al. 2025)
Metabolomics-ML	Random Forests/DNN	TL	Predict phenotypic outputs from metabolomic or fluxomics profiles	Omics data → trait classification/regression	Predicts yeast production traits from metabolomics data.	(Goshisht 2024)
ecFactory	Constraint-based + ML-guided design	D	Predicts optimal metabolic engineering interventions and platform-strain targets	ecYeastGEM + product list → ranked OE/KO/KD gene targets	Designs *S. cerevisiae* strains for 100 + chemicals; prioritizes edits before Build/Test	(Domenzain et al. 2025)
Camformer	Deep CNN for promoters	L→D	Learns promoter→ expression mapping; enables *in-silico* promoter design	Promoter sequences → predicted expression	Trained on millions of *S. cerevisiae* promoters; guides regulatory-part libraries	(Dash and Bornelöv 2025)
Pymaker (pre-trained promoter model)	Transfer learning/foundation DNA model	L→D	Pre-training + fine-tuning to improve promoter activity prediction/design	Unlabeled promoter + labeled yeast data → activity predictor	Improves *S. cerevisiae* promoter design with less data	(Yang et al. 2025)
Codon-choice PLM	Protein language model	L→D	Infers synonymous-codon constraints directly from protein sequence; recommends codon usage	AA sequence → codon choice/optimization signals	Codon optimization for yeast expression driven by PLM-learned constraints	(Sakharova and Lareau 2025)
DLTRNM (distributed large-scale transcriptional regulatory neural network)	Knowledge-integrated ML model	L	Reconstructs transcriptional regulatory networks using pre-trained ML + prior knowledge	Multi-omics + priors → TRN edges & regulators	Maps *S. cerevisiae* regulation to reveal engineering levers	(Fan et al. 2025)
Polygraph	Software framework (ML-assisted assessment)	TL	Systematic evaluation of native and designed promoters	Promoter sequences + models → divergence/feature analyses	Benchmarks computationally designed *S. cerevisiae* promoters prior to build	(Lal et al. 2025)
DL-based yeast colony counter	Deep learning (object detection)	T	High-throughput CFU/colony counting from plate images (species-aware)	Plate images → colony counts & labels	Automates test-stage imaging; distinguishes *S. cerevisiae* vs. *K. humilis*	(Aiki et al. 2025)
ML-assisted signal peptide design (*Y. lipolytica*)	ML-guided sequence design + directed evolution	L→B→T	Optimizes secretion via data-driven SP selection/engineering	SP sequences + secretion data → next-round variants	Boosts heterologous protein secretion in yeast	(Z. Wu et al. 2025)
Automated hypothesis & experiment planner	LLM agent + automated lab	DBTL	Generates hypotheses, plans/executes experiments, logs outcomes	Objective + assay APIs → next experiment + results graph	Demonstrated on *S. cerevisiae* interaction experiments; fits closed-loop biofoundry ops	(Brunnsåker et al. 2025)

### Importance of manually curated data

AI enables powerful data analysis and predictive modelling by identifying complex patterns in large, high-dimensional datasets (Camacho et al. 2018). However, its performance depends heavily on training data quality; biased or sparse datasets can yield unreliable results compared to expert-guided methods (Goshisht 2024). This underscores the need for human–in–the–loop workflows, where domain experts curate training data, validate AI outputs, and provide continuous feedback to refine models. Manually curated data, shaped by expert knowledge, are particularly valuable for rare events or poorly characterized phenomena and often offer greater interpretability when exploring biological mechanisms, as discussed in a preprint (Samek 2017).

Hybrid approaches are emerging to combine the strengths of both. BioNursery, for example, uses LLMs to generate hypotheses, refined via crowd-sourced expert input to avoid hallucinations and ensure relevance (Jamil et al. 2024), while domain-specific tools like CRISPR-GPT and GP-GPT rely on manual verification, (Lyu et al. 2024, Qu et al. 2024).

This integration of AI and expert curation offers a pragmatic middle ground, accelerating discovery without compromising reliability. As data infrastructures mature and AI systems gain self-correction capabilities, the field advances toward fully autonomous biofoundries that execute design, experimentation and learning cycles with minimal human intervention whilst preserving scientific oversight

## Toward autonomous and self-optimizing biofoundries

Automation is central to biofoundries, with fully autonomous, self-optimizing ‘self-driving labs’ integrating robotic execution with AI-driven decision-making to cycle through DBTL phases with minimal human input (Kim 2025). Protein language model-guided systems have autonomously generated enzyme variants, built and tested them, and refined designs over multiple iterations (Zhang et al. 2025), whilst cell-free platforms using a language model and active learning achieved improved protein yields without manual intervention (Herisson et al. 2024).

Advances in hardware, such as automated pickers, fermenters, analysers, and software, including Bayesian optimization and AI-guided design, are accelerating strain engineering. These tools help prioritize informative experiments and avoid unproductive iteration (‘*involution*’) in DBTL cycles (Liao et al. 2022). Early autonomous platforms have shown success in narrow tasks like enzyme optimization; the next step is scaling to whole-cell engineering (Bozkurt et al. 2025).

Importantly, these advances extend beyond *S. cerevisiae*. Modern biofoundries increasingly support other yeasts like *K. phafii* and *Y. lipolytica*, aided by modular toolkits and adaptable automation. Biofoundries have integrated multiple organisms for various applications, though each requires tailored methods. For example, *Y. lipolytica* miniaturized workflows leads to perturbations in phenotype such as flocculation, necessitating hardware innovations (Celińska and Gorczyca 2024).

The effectiveness of autonomous, self-optimizing biofoundries depends on data quality within iterative DBTL cycles. Despite rapid progress, technical bottlenecks constrain full autonomy: high-throughput readouts produce noisy proxy measurements that impede robust model training (Chafai et al. 2023); heterogeneous data integration remains challenged by differing experimental scales, temporal patterns, and batch effects (de Crécy-Lagard et al. 2025). Hardware reliability issues, including robot failures, and inter-instrument differences, impede large-scale data generation and sharing (Kim et al. 2025, Tobias and Wahab 2025). Optimization landscapes for whole-cell engineering are high-dimensional and rugged; strategies that work for low-dimensional enzyme optimization do not yet scale efficiently to multi-trait, environment-dependent cellular phenotypes within affordable experimental budgets (Aghdam et al. 2024). Finally, insufficient provenance and metadata prevent reliable federated learning across facilities, limiting sample diversity for robust model generalization. Advancing autonomous biofoundries therefore requires concurrent improvements in measurement science, instrument standardization, robust ML methods for distribution shift, and richer, large multi-modal datasets.

Beyond technical challenges, economic costs and human capital present additional barriers. Establishing a modern biofoundry requires substantial capital expenditure for automation, instrumentation, and compute infrastructure; operating costs and high-availability staffing elevate total cost of ownership (Vickers and Freemont 2022, Asin-Garcia et al. 2024). Capital intensity and uncertain return timelines deter academic groups and SMEs from investing in in-house biofoundries, driving reliance on shared facilities (Vickers and Freemont 2022). Although automation reduces demand for traditional wet-lab expertise, biofoundries require a fundamentally different skill set: computational biologists, automation engineers, and data scientists. Current gaps in AI literacy, systems-level automation, and data engineering reflect a mismatch between available skills and those demanded by autonomous DBTL workflows (Trevisan et al. 2024). These barriers argue for policy interventions (shared-facility funding, subsidized training pipelines), standardized modular equipment to reduce entry costs, and open curricula building an ‘AI-ready’ biotech workforce.

## Standardization for reproducibility

While bespoke protocols produce reliable data within a single biofoundry, robust standardization of assay methods, data schemas, and metadata ontologies are critical for sharing and aggregating datasets across facilities, a prerequisite for training generalizable AI models. Pre-defined, validated workflows and uniform file formats also reduce lead times for new experiments by eliminating bespoke method development (Casas et al. 2024, Kim et al. 2025). Without harmonization, cross-site integration stalls and automated DBTL efficiency is severely limited.

Biofoundries advance standardization through modular, automated workflows that reduce human error (Gupta et al. 2024). Toolkits like YeastFab, Yeast Optogenetic Toolkit, and Multiplex Yeast Toolkit have improved DNA assembly efficiency (Shaw et al. 2023, Harmer and McClean 2025, Hoffmann 2025), though variations in enzymes, reaction conditions, and equipment calibration still affect outcomes, highlighting the need for benchmarking across diverse chassis organisms.

Phenotypic screening encompasses assays tailored to the trait under investigation: growth kinetics, product titres, reporter-based fluorescence, cell viability, enzyme activity, and metabolite quantification (Pham et al. 2021). Core protocol elements such as media composition, pH, inoculum density, temperature, and calibration workflows should be standardized to ensure comparability across experiments and laboratories (Lloyd 2020). Conversely, bespoke methods remain necessary for novel measurements such as custom droplet-based assays or specialized LC-MS/MS workflows, where detection chemistries and instrument parameters vary with each target. Differences in statistical analysis approaches and proprietary software further undermine cross-study comparability (Gilbertson et al. 2024). Reproducibility therefore requires harmonization of core assay conditions and data-processing pipelines for routine traits, alongside clearly documented custom protocols for specialized measurements.

Community standards are advancing standardization. SBOL enables genetic design exchange, whilst repositories like BioBrick, SEVA, and SynBioHub support part reuse (Smolke 2009, Chao et al. 2017, Beal et al. 2020). Open-source platforms such as Synthace and OpenWorkstation encapsulate validated protocols in programmable formats (Anhel et al. 2023, Torres-Acosta et al. 2024), while MQTT-based systems and Python APIs support cross-platform calibration (Eggert et al. 2020, Gervasi et al. 2021).

Standardized phenotypic metrics, such as TRY, are also gaining traction (Beal et al. 2020). As biofoundries generate increasingly large datasets, alignment on QC thresholds, metadata standards, and findable, accessible, interoperable, reusable (FAIR) data principles becomes crucial for enabling machine learning and cross-site validation (Pleiss 2024).

To move from principles to practice, we recommend an evidence-backed harmonization roadmap that leverages existing community standards and GBA coordination. Immediately actionable steps include: (1) adopt common exchange formats and ontologies across DBTL phases (e.g. SBOL v3 for design, COMBINE/OMEX for model, and simulation packaging, MIxS/GenBank for sequence deposition and MIFlowCyt/MIQE/MIAPE for assay metadata where applicable) to ensure machine-readable interoperability and re-use; (2) require ‘minimum information’ checklists for DNA constructs on publication, building on proposals for complete sequence disclosure, machine-readable files, and provenance metadata to enable benchmarking and replication; (3) define a focused, publishable minimal reporting standard for DNA assembly and genome editing experiments to be endorsed by journals and funders, capturing full sequences, assembly methods, fragment conditions, QC methods and readouts, and strain deposition identifiers; (4) implement cross-location benchmarking exercises and ring-trials coordinated by the Global Biofoundry Alliance to quantify inter-lab variance for core assays and publish standard operating ranges and QC thresholds; (5) mandate persistent identifiers and centralized deposition (Addgene/PIPdb/SynBioHub/ENA) for constructs and metadata with machine-readable schemas (JSON-LD or SBOL) to support automated aggregation; (6) promote open reagent and enzyme formulation reporting so that enzymatic sources are not hidden variables in transferability; and (7) couple technical steps with capacity building, coordinated training modules, published protocol libraries in executable formats (Autoprotocol/OPIL/SBOL), and a shared hardware calibration package to reduce entry costs for new sites. Many elements align with recent community efforts: SBOL v3 provides a robust design exchange format, and coordinated benchmarking has shown that systematic harmonization materially reduces variance between laboratory locations (Hillson et al. 2019, McLaughlin et al. 2020, Bock et al. 2022, Thuronyi et al. 2023, Claussnitzer et al. 2024, Golebiewski et al. 2024, Vegh et al. 2024). Operationalizing these steps will require coordinated endorsement by the Global Biofoundry Alliance, major funders and journals, coupled with modest investments in tooling for metadata capture and automated deposition to reduce compliance barriers for experimental teams.

### DNA assembly: advances and standardization challenges

DNA assembly methods like Golden Gate (Engler et al. 2008, Lee et al. 2015) and Gibson Assembly (Gibson et al. 2009) underpin HTP DBTL cycles in yeast engineering. Modular Golden Gate–based toolkits, including the scarless Golden EGG system (Biró et al. 2024), and a MoClo–compatible plasmid set for hierarchical assembly were created (de Vries et al. 2025). Integration with automation platforms, such as AssemblyTron on Opentrons OT–2 (Bryant et al. 2023) and PlasmidMaker iBioFAB (Enghiad et al. 2022), enables parallel construction of thousands of constructs with minimal manual intervention. Yeast’s native homologous recombination simplifies large fragment assembly, enabling entire synthetic chromosomes like synXVI (>900 kb) (Goold et al. 2025).

Enzymatic DNA synthesis delivers kilobase fragments with gene–scale assembly compatibility (Simmons et al. 2023). HTP liquid–handling platforms integrates one–pot HiFi/Type IIS reactions, generates thousands of constructs per day with error frequencies below one per 10 kb  (Ma et al. 2024). Continuous–flow microfluidic systems miniaturize cell–free assemblies to nanolitre volumes, cutting reagent costs by two orders of magnitude (Baranwal and Maerkl 2024). Updated multi–kingdom Golden Gate toolkits define consensus overhang sets and reaction buffers that increase cross–lab success rates, and modular design pipelines now feed directly into robotic execution (Vegh et al. 2025). Commercial bench–top printers such as the BioXp 3250 bring chip–based oligo synthesis and gene assembly together, shortening design–to–build cycle to one day (Ma et al. 2024). Complementary standards like SEVA 4.0 provide curated vectors with harmonized origins and selection markers to enhance plug–and–play interoperability (Martínez-García et al. 2023).

Reproducibility remains a bottleneck: Inter–lab comparisons show order–of–magnitude differences in Golden Gate yields due to proprietary enzyme blends and undocumented buffer additives (Bell and Molloy 2022). Overhang sequence context can halve efficiency, underscoring the need for agreed design rules and minimum-information checklists capturing enzyme provenance, ligation kinetics and fragment purity (Lux et al. 2023, Strzelecki et al. 2024). Comprehensive user guides have therefore recommended minimum–information checklists that capture enzyme provenance, ligation kinetics and fragment purity to facilitate benchmarking (Bird et al. 2022). Another study highlighted that despite automated protocols, construct design choices strongly influence cross–facility performance (Cummins et al. 2023). Calls for stronger, simpler publishing standards argue that complete sequence disclosure and open reagent formulations are prerequisites for scalable, community–wide validation (Thuronyi et al. 2023). In practice, journals and community started operationalizing these ideas: A proposed mandatory sequence deposition and a short ‘Box 1’ style minimum reporting checklist for DNA constructs (report full sequences in GenBank/FASTA, note which parts are empirically validated, deposit physical samples where possible), and more specialized minimum-information standards (for example, MAVE and pooled-CRISPR reporting guidelines) show how compact, targeted checklists can accelerate reuse of DNA construct designs, sequence-resolved datasets, and associated phenotype measurements in benchmarking, meta-analysis, and ML applications (Bustin et al. 2009, Bock et al. 2022, Claussnitzer et al. 2024).

### Genome editing advances and standardization challenges

Genome editing in yeast has diversified beyond CRISPR–Cas9 to include a range of novel nucleases and precision–editing modalities. CRISPR–Cas system remains the foundation for most workflows in *S. cerevisiae*, praised for its simplicity, high targeting specificity, and operational efficiency (X. Wu et al. 2025). Alternative nucleases such as ErCas12a offer T–rich PAM recognition and near–100% editing efficiencies (Bennis et al. 2023). Base editors have also been engineered in yeast, including a first–of–its–kind diversifying cytosine deaminase (AID) fusion that enables rapid C–to–T transitions without double–strand breaks (Cazier et al. 2023). Multiplexed editing combining optimized sgRNA arrays and high–throughput transformation protocols have reduced strain engineering timelines from weeks to days (Zhang et al. 2021). A Cas9–fusion strategy (Cas9–Brex27–FadR) recruits achieved integration efficiencies of 98% for 10 kb fragments and nearly 80% for 40 kb fragments (Xu et al. 2024). For even larger assemblies, the HAnDy protocol employs CRISPR–assisted chromosome elimination enables Mb–scale pathway engineering (Ma et al. 2025).

An emerging alternative to conventional editing is the *de novo* synthesis and deployment of entire synthetic chromosomes. In November 2023, the Sc2.0 consortium reported a strain containing more than 50% synthetic DNA across seven artificial chromosomes, demonstrating that cells tolerate extensive genome rewiring (Goold et al. 2025). Subsequent efforts have consolidated multiple synthetic chromosomes into single strains, revealing novel genetic interactions and streamlined strain backbones for reproducible engineering (Zhao et al. 2023).

To date, the most pressing obstacle is cross–lab reproducibility. Comparative studies show that variations in sgRNA scaffold design, Cas expression vectors, and donor–template architecture can shift editing efficiencies by an order of magnitude, highlighting the need for harmonized reporting standards (Antony et al. 2022). Recent reviews echo this concern and propose minimum–metadata checklists, covering PAM context, off–target assays, and transformation parameters, to enable meaningful benchmarking across platforms (X. Wu et al. 2025). For genome editing, community recommendations provide templates that can be adapted into a compact ‘Minimum Information for Genome Editing’ checklist covering sgRNA sequences and scaffolds, delivery vectors and expression context, donor template sequence and homology arms, off-target assay methods and sensitivity, transformation efficiencies, and deposition of editing reagents/cells to repositories (Beal et al. 2020, Bock et al. 2022, Malci et al. 2022, Skrekas 2022, Thuronyi et al. 2023, Claussnitzer et al. 2024). Formalizing these elements as a field-endorsed minimum standard, and linking compliance to routine deposition in public repositories, would substantially reduce ambiguity in cross-facility comparisons and enable automated QC in biofoundry networks.

### Standards and metrics for reproducibility and interoperability

Achieving reliable, scalable bioengineering requires community standards for data, methods, and performance metrics (Hillson et al. 2019, Beal et al. 2020). Over the last few years, there has been a strong push toward technical standards that promote reproducibility and interoperability in synthetic biology. One example is the Synthetic Biology Open Language (SBOL), which has been updated (v3.0/3.1) to facilitate exchanging genetic designs and engineering intent across software platforms (Quinn et al. 2015, Vidal et al. 2025). SBOL provides a structured format to describe DNA parts, circuits, and assemblies, enabling design compatibility across laboratories and automation systems.

Standard ontologies and COMBINE archives are increasingly adopted to package datasets, models, and metadata together, ensuring consistent interpretation and enabling data-centric automation (Matzko and Konur 2024). These structured formats form a ‘digital language’ for biofoundries, aligning design environments with build and test systems across institutional boundaries.

International consortia, academic institutions, industry partners, and regulatory bodies all recognize the need for unified standards. The Global Biofoundry Alliance (GBA) supports international collaboration by promoting standardized workflows, data formats, and best practices (Hillson et al. 2019). Similarly, the International Genetically Engineered Machine (iGEM) Competition reinforces standardized design principles, contributing over 20 000 modular parts to the Registry of Standard Biological Parts and training thousands of researchers (Smolke 2009, Gupta et al. 2024).

Recent studies highlight the importance of performance metrics and data quality control in synthetic biology. For instance, a remote robotic lab replicated a classic yeast gene circuit experiment at scale, revealing which results remained robust under HTP scrutiny (Golebiewski et al. 2024). Standard performance metrics such as TRY are now commonly used to evaluate industrial strain development and bioprocess viability.

Another key technical focus is laboratory interoperability. Efforts from other fields, such as medical imaging’s DICOM standard, have inspired sharable protocol formats and toolchains. Frameworks such as MoClo, Phytobricks, and Loop provide standardized DNA assembly methods compatible with automation platforms (Vidal et al. 2025). Open-access repositories like Addgene, SEVA, and SynBioHub facilitate access to validated plasmids, strains, and parts (Vegh et al. 2025).

Ultimately, standardization of methods, formats, and metrics is driving a new era of collaborative synthetic biology. As biofoundries proliferate worldwide, they rely on common data structures, interoperable instrumentation, and community-wide best practices. These foundations enable distributed DBTL workflows, where design, build, and test operations occur in different institutions without the need for extensive reformatting or re-validation. By ensuring experiments can be repeated, improved, and transferred with confidence, standardization becomes a cornerstone of scalable, reliable bioengineering.

## Policy, regulatory, and data governance for global biofoundries

As biofoundries expand globally, policy and governance frameworks are evolving to support safe, collaborative, and efficient development. A central focus is data governance: protocols for sharing designs, metadata, and materials enable reproducibility across labs (Asin-Garcia et al. 2024, Watkins et al. 2024). A yeast strain producing a novel antibiotic, for instance, can be transferred between facilities if both adhere to shared standards such as SBOL and minimal phenotypic datasets. This effort also includes open MTAs and cloud-based experimental logging systems, like those from the DARPA SD2 program (Leins et al. 2023).

Standardized phenotypic screening and quality metrics are critical for HTP workflows (Tancheva et al. 2025). Researchers are aligning assay conditions, media, and reporter systems (Beal et al. 2020, John et al. 2020), while guidelines like MIACA, and FAIR principles promote transparency (Nault et al. 2023). Platforms such as protocols.io and FAIRDOMHub facilitate method sharing and reduce variability (Gygli and Pleiss 2020, ‘Welcoming protocols.io,’ 2024). Reporting negative results is also encouraged to support iterative improvement (Brazil 2024).

Regulatory landscapes increasingly shape foundry operations. Australia’s Gene Technology Regulations 2001 exempt SDN1 edits from GMO oversight, directly affecting strain design strategies and regulatory data packages; however, this exemption is sometimes misrepresented, prompting calls for precise regulatory mapping in distributed DBTL programs (Thygesen 2024). China’s Biosafety/Biosecurity Law (2020/2021) consolidates governance of biotechnology R&D, human genetic resources, and pathogen laboratories, while analyses note persistent implementation gaps relevant to distributed foundry networks (Cao 2021). Singapore’s risk-based system and investments in SynCTI and SINERGY illustrate a supportive innovation ecosystem attentive to dual-use concerns and interoperable data sharing (Trump et al. 2020, Farzaneh and Freemont 2021, Millett et al. 2023). Comparative analyses show China pairing statutory controls with soft-law frameworks such as the Tianjin Biosecurity Guidelines, whereas Singapore integrates infrastructure and governance to support regional DBTL interoperability (Trump et al. 2020, Hynek 2025).

Technical standards are increasingly tied to regulatory compliance, especially as biofoundries move toward deployment phases (Freemont 2024). ISO Technical Committee 276 is drafting guidelines on bioprocessing and data traceability, supported by efforts from metrology institutes like NIST (Farzaneh and Freemont 2021). Biofabrication standards, paralleling GMP, aim to ensure consistent yields and reliable data across labs (Beal et al. 2020, Vidal et al. 2025). Internationally, biosafety oversight remains uneven: ISO 35 001:2019 offers the first global biorisk-management standard, but it is still not widely adopted, and many regions continue to rely on BMBL or the WHO Laboratory Biosafety Manual; frameworks that currently omit explicit AI safeguards (Undheim 2024).

With AI-driven automation, biosecurity and ethics are becoming more pressing. A 2024 publication portrays the situation as a ‘whack-a-mole’ governance challenge, advocating a dynamic blend of precautionary hard law, stewardship-oriented soft law, bottom-up community norms, and industry-led initiatives to keep risks in check (Undheim 2024).

A multi-stakeholder red-team study demonstrated that generative protein design tools could produce synthetic homologs that initially evaded standard sequence-order screening (Wittmann et al. 2025). Following responsible disclosure, industry screening systems were patched to ∼97% detection accuracy, restricting ability to order genes encoding AI-designed risky proteins. Separately, public LLMs show tightening safeguards: certain models refused disinformation prompts even under jailbreaking, whilst others improved safeguards over time, indicating operational restrictions applied by AI developers relevant to bioscience communication risk (Menz et al. 2024). Policy scholarship argues for mandatory evaluations of biological foundation models, linking general-purpose AI regulation to proposed model-level safeguards and controlled access to AI-enabled biological design (Grinbaum and Adomaitis 2024).

Beyond design and predictive modelling, specialized workflows extend into gene editing, genotype–phenotype mapping, and biosafety. GP-GPT captures genotype–phenotype links across large datasets (Lyu et al. 2024), whilst ThreatSeq screens for potentially hazardous sequences in real time to strengthen sequence-level biosurveillance (Hoffmann et al. 2023). Deploying protein language models at scale depends on consistently labelled training data and integration with robust laboratory information management systems synchronized with automated build–test pipelines (Cheng et al. 2023, Song et al. 2024). Regulators increasingly emphasize traceability and interpretability in AI-driven experimentation; early engagement is prudent because LLMs and generative models can propose constructs outside typical design boundaries (Boiko et al. 2023). Proposals include screening AI-generated designs and tracking engineered organisms to prevent misuse (Holub and Agena 2023). Data ownership and privacy have emerged as central sociolegal concerns in collaborative synthetic biology as the balance between openness and commercial becomes more relevant (McLennan and Maslen 2025). Federated data trust architectures and controlled access repositories using attribute-based access controls and blockchain-enabled provenance tracking are being implemented to secure data sharing whilst preserving intellectual property rights (Taddese et al. 2025). Targeted training initiatives including embedded science data literacy programs and interdisciplinary workshops have been incorporated into graduate and professional curricula to equip scientists with competencies in data curation, ethical sharing and regulatory compliance (Qiao et al. 2024).

Automation tools further reinforce standardization. Platforms like AssemblyTron, PyLabRobot, and DNAda translate genetic designs into reproducible liquid-handling protocols with built-in monitoring (Bryant et al. 2023, Nava et al. 2023, Wierenga et al. 2023). In a recent preprint, RoboCulture, an AI-driven, hardware-agnostic system enhances portability across robotic setups (Angers et al. 2025), while benchmarking tools support strategic automation choices (Rupp et al. 2024). These systems align with FAIR principles and improve traceability (Marilene et al. 2021, Pleiss 2024).

Global interoperability depends on shared standards and mutual trust. The UK, Singapore, and China are building compatible infrastructures where design, testing, and analytics can be distributed across locations (Asin-Garcia et al. 2024, Watkins et al. 2024). Regulatory bodies are updating submission protocols to track strains developed through automated cycles, with blockchain-style traceability and kill switch safeguards under discussion (Farzaneh and Freemont 2021). Emerging frameworks call for AI-enabled early-warning systems to detect and mitigate biohazards and for broadening the Dual Use Research of Concern (DURC) regime so that generative-AI design tools fall within its scope. Multi-level governance continuums, spanning global, national, corporate, laboratory, and citizen tiers, are being proposed to ensure that precautionary, stewardship, bottom-up, and market-driven measures act in concert (Undheim 2024).

In summary, data governance and policy are key to scaling the biofoundry revolution. Shared protocols, robust quality metrics, and strong biosecurity frameworks are enabling reproducible, distributed, and responsible innovation (Hillson et al. 2019, Leavell et al. 2020). Through global harmonization of DBTL workflows, synthetic biology is transitioning from *ad hoc* experimentation to a coordinated, scalable, and secure engineering discipline (Holub and Agena 2023).

## Cross-domain inspirations: learning from materials and pharma

Synthetic biology increasingly draws on advances from adjacent fields like nanomaterials, chemical engineering, and drug discovery. Self-driving labs, originating in chemistry and materials science, employ closed-loop systems where robots run experiments and AI selects the next ones, accelerating discoveries in catalysts and light-harvesting materials (Martin et al. 2023, Kim 2025). Bioengineers are adapting similar approaches, using Bayesian optimization and evolutionary algorithms, first popular in drug formulation, to guide strain engineering by selecting gene combinations or culture parameters.

AlphaFlow, an AI-controlled chemical synthesis system, parallels biofoundry pipelines where multiple reactions or design steps must be orchestrated (Volk et al. 2023). Lessons from AlphaFlow’s decision logic can inform control systems for complex metabolic engineering. Similarly, HTS in pharma, used to test millions of compounds, is analogous to testing large strain libraries or combinatorial pathways in synthetic biology (Salunke et al. 2022). Drug discovery workflows increasingly rely on *in silico* design and cloud labs, like IBM’s RoboRXN, where users specify outcomes and automation handles the rest (O’Neill 2021). A similar model is emerging for biofoundries: users could submit a target compound and organism and receive a custom-engineered yeast strain. Standards from pharmaceutical biotechnology, such as SiLA 2 and ANSI/ISA protocols, are being adopted to improve device interoperability (Juchli 2022). Even MLOps, Machine Learning Operations from the software industry, is being applied to maintain and update predictive models used in biofoundry workflows (Alla and Adari 2020).

Regarding materials informatics, active learning techniques used to optimize carbon nanotube production are now applied to protein engineering and metabolic pathway optimization (B. Yang et al. 2022). Digital twins, computational models that simulate experiments, are already being used in synthetic biology to model bioreactors or predict metabolic outcomes, reducing trial-and-error in the Design phase (Martin et al. 2023).

Operational practices from semiconductor manufacturing and industrial automation also offer valuable lessons. Cleanroom protocols, QA/QC procedures, and continuous improvement philosophies (kaizen) are being adopted to enhance biofoundry reproducibility (Chien et al. 2020, Zhao and Yoshikuni 2021). Similarly, pre-competitive collaborations in pharma and materials science, like the Materials Genome Initiative, have inspired data-sharing efforts such as the Global Biofoundry Alliance (Holub and Agena 2023).

Regulatory insights from other domains, such as FDA oversight of automated drug production, are shaping discussions about oversight for engineered microbes produced by biofoundries (Niazi 2023). Synthetic biology is not evolving in isolation but converging with automation, AI and data science. This cross-pollination is making biofoundries more efficient and integrated by design.

## Conclusion

High-throughput yeast engineering is shifting from labour-intensive trial-and-error to AI-driven, automated platforms that can design, build, test, and learn at unprecedented speed. Biofoundries are no longer just tools to increase throughput; they are cyber-physical infrastructures transforming how we prototype and deploy biological systems. As synthetic biology advances, the convergence of robotics, machine learning, and multi-omics is enabling truly autonomous, self-optimizing ‘self-driving labs.’ Yet technology alone is not enough. Scalable and responsible innovation requires standardization, robust data governance, and harmonized policy frameworks.

Governance must evolve alongside capability. The 1975 Asilomar conference on recombinant DNA set in progress proactive, precautionary frameworks, showing that transparency and regulation can enable innovation while safeguarding society. At the 2025 Asilomar conference on the future of biotechnology, a range of topics were discussed (resulting entreaties are available at https://repository.rice.edu/collections/e4e3a33e-08a4-427a-9b59-cb30d93b8bbe), including around AI-driven design and biosafety. Embedding auditability, explainability, and robust oversight into biofoundry operations will be essential to maintain public trust as the scale and autonomy of these systems increase.

Equitable access is also central to the long-term impact of high-throughput yeast engineering. Without deliberate investment in shared tools and distributed capacity, advanced workflows risk remaining concentrated in countries with existing research strengths. Initiatives that work on open-source biotechnology and local biomanufacturing illustrate how this can be addressed—through open enzyme collections, community-driven reagent production, and training programmes that lower barriers to participation. These examples demonstrate that equitable access is not peripheral but foundational, ensuring that biofoundries evolve from elite infrastructures into global engines of collaboration and innovation.

The next generation of yeast bioengineering will be defined not only by smarter tools but by a smarter system. Biofoundries are becoming the operating system for a new era of biological design; one that demands coordinated investment in automation, AI, and governance while embedding transparency, equity, and sustainability at its core.

## Supplementary Material

foag003_Supplemental_File
