# Supplementary material for: High-throughput yeast engineering in biofoundries: towards autonomous and scalable synthetic biology
Source: FEMS Yeast Res. 2026 Jan 27;26:foag003. doi: 10.1093/femsyr/foag003 (PMC12927428; doi:10.1093/femsyr/foag003)
Supplement: foag003_Supplemental_File [file foag003_supplemental_file.docx]

*Table S1. Artificial intelligence tools with demonstrated utility in other systems but not yet validated in yeast engineering.*

| ****TOOL / MODEL**** | ****TYPE**** | ****DBTL ROLE**** | ****FUNCTION / APPLICATION**** | ****INPUT / OUTPUT**** | ****YEAST ENG. APPLICATION**** | ****REFERENCE**** |
| --- | --- | --- | --- | --- | --- | --- |
| **GP-GPT** | **Genotype–Phenotype LLM** | **L** | **Embedding of DNA features, generation of predictive genotype-phenotype models** | **DNA sequences, features - Functional predictions** | **Maps yeast genetic constructs to production phenotypes.** | **(Lyu et al., 2024)** |
| **CRISPR-GPT** | **Workflow-integrated LLM** | **B** | **sgRNA design, off-target prediction, lab automation scripting** | **Genome + PAM rules → sgRNA library + protocols** | **Automates multiplex guide‑RNA design for genome editing.** | **(Qu et al., 2024)** |
| **ACTIVE LEARNING LOOPS** | **ML strategy** | **TL** | **Closed-loop data collection and model refinement for optimisation** | **Design space + experimental data → Next experiment** | **Iteratively refines experiments to maximise yields.** | **(Yang et al., 2022)** |
| **BAYESIAN OPTIMIZATION** | **ML strategy** | **L** | **Suggests next-best experiment for yield maximisation based on uncertainty** | **Experimental history → Suggest next inputs** | **Recommends the most informative yeast experiments for optimisation.** | **(Martin et al., 2023)** |
| **TAPE (Transformer)** | **DNA/RNA/Protein transformer** | **DL** | **Encoding biological sequences for regression/classification tasks** | **Sequences → Expression / activity scores** | **Generates embeddings of sequences for predictive models.** | **(Rao et al., 2019)** |
| **SCVI / VAE Models** | **Deep generative models** | **L** | **Dimensionality reduction of single-cell data; identification of rare phenotypes** | **scRNAseq → Clustered phenotypes** | **Identifies rare cell subpopulations for targeted engineering.** | **(Lopez et al., 2018)** |
| **LLM-RDF (GPT-4 Backend)** | **Multi-agent LLM framework** | **DBTL** | **End-to-end orchestration of chemical synthesis via LLM agents** | **Natural language + context → Workflow actions** | **Converts strain design goals into DBTL protocols.** | **(Ruan et al., 2024)** |

Lopez, R., Regier, J., Cole, M. B., Jordan, M. I., & Yosef, N. (2018). Deep generative modeling for single-cell transcriptomics. *Nature methods*, *15*(12), 1053-1058.

Lyu, Y., Wu, Z., Zhang, L., Zhang, J., Li, Y., Ruan, W., Liu, Z., Yu, X., Cao, C., & Chen, T. (2024). Gp-gpt: Large language model for gene-phenotype mapping. *arXiv preprint arXiv:2409.09825*.

Martin, H. G., Radivojevic, T., Zucker, J., Bouchard, K., Sustarich, J., Peisert, S., Arnold, D., Hillson, N., Babnigg, G., & Marti, J. M. (2023). Perspectives for self-driving labs in synthetic biology. *Current opinion in biotechnology*, *79*, 102881.

Qu, Y., Huang, K., Cousins, H., Johnson, W. A., Yin, D., Shah, M., Zhou, D., Altman, R., Wang, M., & Cong, L. (2024). CRISPR-GPT: An LLM Agent for Automated Design of Gene-Editing Experiments. *bioRxiv*. <https://doi.org/10.1101/2024.04.25.591003>

Rao, R., Bhattacharya, N., Thomas, N., Duan, Y., Chen, P., Canny, J., Abbeel, P., & Song, Y. (2019). Evaluating protein transfer learning with TAPE. *Advances in neural information processing systems*, *32*.

Ruan, Y., Lu, C., Xu, N., He, Y., Chen, Y., Zhang, J., Xuan, J., Pan, J., Fang, Q., & Gao, H. (2024). An automatic end-to-end chemical synthesis development platform powered by large language models. *Nature communications*, *15*(1), 10160.

Yang, H., Li, J., Lim, K. Z., Pan, C., Van Truong, T., Wang, Q., Li, K., Li, S., Xiao, X., & Ding, M. (2022). Automatic strain sensor design via active learning and data augmentation for soft machines. *Nature Machine Intelligence*, *4*(1), 84-94.
